# Production and Characterization of a Bioflocculant Produced by *Bacillus* *salmalaya* 139SI-7 and Its Applications in Wastewater Treatment

**DOI:** 10.3390/molecules23102689

**Published:** 2018-10-18

**Authors:** Zayed M. Abu Tawila, Salmah Ismail, Arezoo Dadrasnia, Mohammed Maikudi Usman

**Affiliations:** 1 Institute of Biological Science, Faculty of Science, University of Malaya, Kuala Lumpur 50603, Malaysia; zabutawila@gmail.com (Z.M.A.T.); are.dadrasnia@gmail.com (A.D.); mmusumanu@gmail.com (M.M.U.); 2 Department of Biology, Faculty of Science, Al-Azhar University, Gaza, Palestine

**Keywords:** *Bacillus salmalaya*, bioflocculant, optimization, production, characterization, wastewater treatment

## Abstract

The production, optimization, and characterization of the bioflocculant QZ-7 synthesized by a novel *Bacillus salmalaya* strain 139SI isolated from a private farm soil in Selangor, Malaysia, are reported. The flocculating activity of bioflocculant QZ-7 present in the selected strain was found to be 83.3%. The optimal culture for flocculant production was achieved after cultivation at 35.5 °C for 72 h at pH 7 ± 0.2, with an inoculum size of 5% (*v*/*v*) and sucrose and yeast extract as carbon and nitrogen sources. The maximum flocculating activity was found to be 92.6%. Chemical analysis revealed that the pure bioflocculant consisted of 79.08% carbohydrates and 15.4% proteins. The average molecular weight of the bioflocculant was calculated to be 5.13 × 10^5^ Da. Infrared spectrometric analysis showed the presence of carboxyl (COO-), hydroxyl (-OH), and amino (-NH_2_) groups, polysaccharides and proteins. The bioflocculant QZ-7 exhibited a wide pH stability range from 4 to 7, with a flocculation activity of 85% at pH 7 ± 0.2. In addition, QZ-7 was thermally stable and retained more than 80% of its flocculating activity after being heated at 80 °C for 30 min. SEM analysis revealed that QZ-7 exhibited a clear crystalline brick-shaped structure. After treating wastewater, the bioflocculant QZ-7 showed significant flocculation performance with a COD removal efficiency of 93%, whereas a BOD removal efficiency of 92.4% was observed in the *B. salmalaya* strain 139SI. These values indicate the promising applications of the bioflocculant QZ-7 in wastewater treatment.

## 1. Introduction

Flocculants are macromolecules with the ability to flocculate suspended solids, cells, and solid colloid particles [1]. Flocculants are widely utilized in separation techniques, such as in drinking water purification, wastewater treatment, activated sludge dehydration, downstream processing, and food fermentation [2]. Flocculants are typically classified into three groups: synthetic organic flocculants such as polyethyleneimine and polyacrylamide byproducts, inorganic flocculants, including aluminum sulfate and polyaluminum chloride, and natural flocculants (bioflocculants) such as chitosan sodium alginate [3].

Bioflocculation is defined as a process in which mediation of flocculants is achieved in the presence of microorganisms or biodegradable macromolecular flocculants released by microorganisms [4]. A number of microorganisms (i.e., bacteria, algae, fungi, and actinomycetes) are considered producers of bioflocculants [5]. Bioflocculants such as diatom silica shells and *Arthrobacter* spp. biomass are applicable in removing heavy metals, including arsenate, from wastewater [6,7]. Bioflocculation is considered an active process caused by living cells due to the production of exopolymeric macromolecules. The flocculation process in microorganisms was first reported in yeast by Louis Pasteur in 1876 [2]. Microbial biopolymers have gained attention as anti-bacterial, anti-viral and anti-algal agents. In addition, they are considered inducers of microbial aggregation and biofilm formation. Moreover, microbial flocculants are also applied in different industries such as the food and pharmaceutical industries as viscosifying, emulsifying, and stabilizing agents, for the purification of potable water, and wastewater treatment [8]. Bioflocculants have been used as biosorbents for removing all types of metallic pollutants from manufacturing wastes [9]. Flocculation and coagulation processes are cost-effective methods used as primary treatment for drinking water [10]. Many inexpensive chemical compounds with high flocculation properties, such as ferric chloride, polyacrylamide, and polyaluminum chloride, are extensively used in such treatments [11]. Zhang et al. [3] reported that some flocculants pose a risk to human health; for example, aluminum salts are associated with Alzheimer’s disease. Moreover, monomeric units of acrylamide can lead to severe neurotoxic and carcinogenic effects. Zhang et al. [3] indicated that acrylamides are nondegradable in nature, and the use of these flocculants is now limited or banned in several countries. Due to their biodegradability and nontoxicity for the environment and human health, bioflocculants could be an alternative to inorganic and synthetic organic flocculants [4,12]. However, despite the fact that bioflocculants of natural origin are biodegradable and safe in application, they often exhibit inadequate flocculating activity [13]. Therefore, the search for new bioflocculants that are both biodegradable and display powerful flocculation capability is attracting much research attention. Various bioflocculants can be derived from different bacteria; the produced bioflocculants can be optimized and characterized. For instance, *Bacillus licheniformis* [14], *Nocardia amarae YK1* [15], *Pacilomyces* sp. [13], and *Rhodococcus erythropolis* S-I [16] all produce flocculating proteins. Species that produce polysaccharide bioflocculants include *Bacillus subtilis* IFO3335 [17] and *Alcaligenes latus* KT201 [18], while *Arathrobacter* sp. [19] and *Arcuadendron* sp. TS-4 [20] produce glycoprotein bioflocculants.

Members from the genus *Bacillus* belonging to the phylum Firmicutes are classified as Gram-positive and spore-forming bacteria with a rod-shaped structure. *Bacillus* species can tolerate facultative anaerobes or obligate aerobes. They comprise naturally ubiquitous pathogenic and free-living species [21]. Moreover, various industrially important species from this genus have a reported history of safe applications in both the pharmaceutical and food industries [5]. For instance, *Bacillus amyloliquefaciens*, *Bacillus clausii*, *B. licheniformis*, *Bacillus megaterium*, and *B. subtilis* were reported to have several advantages in industrial applications [22]. In agricultural biotechnology, many *Bacillus*-derived products serve as microbial biofertilizers, fungicides, or pesticides [23]. *Bacillus amyloliquefaciens* is identified for the synthesis of a ribonuclease (an antibiotic protein occurring in nature), α-amylase, and proteases protein synthesis [24]. The main factors preventing their wider production and industrial application include low yields, high cost of production, and low flocculating activity [25,26]. Therefore, screening and identifying new microorganisms that produce bioflocculants, as well as h programs for the optimization of fermentation conditions, that can help enhance bioflocculant yield are important objectives [27,28]. Industrial wastewater treatment is a hot research topic worldwide, and flocculation is considered a remarkable method for removing pollutants from wastewater [29].

The present study aimed to investigate the potential of *Bacillus salmalaya* strain 139SI to produce bioflocculants, as well as to optimize and characterize the produced bioflocculant QZ-7. The study also determined the ability of the *B. salmalaya* strain 139SI to remove organic matter measured as biological oxygen demand (BOD) and the newly produced bioflocculant QZ-7 to remove organic matter measured as chemical oxygen demand (COD) from wastewater.

## 2. Results and Discussion

### 2.1. Selection of the Bacterial Strain for Bioflocculant Production

Bacterial strains were screened for bioflocculant production. From 15 colonies, five strains presented the highest flocculating activity as shown in Table 1. The maximum flocculating activity was 83.3% for *B. salmalaya* strain 139SI-7. This selected strain was tested for the optimization of bioflocculant production.

### 2.2. Optimization of B. salmalaya Strain 139SI-7 for Bioflocculant Production

#### 2.2.1. Effect of pH

**Figure 1:**
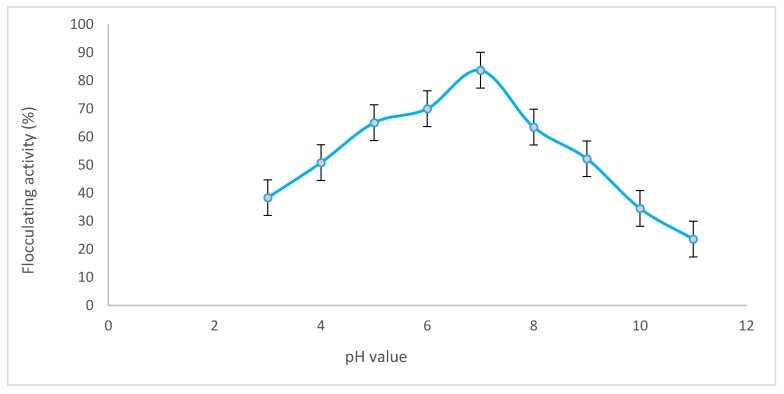
Effect of pH on bioflocculant production.

The initial pH of the fermentation medium directly influenced the synthesis of the bioflocculant QZ-7. Flocculating activity in the fermentation broth culture start gradually increased from 38.6% at pH 3 to 83.7% at pH 7 and gradually decreased to 28.8% at pH 11 as shown in Figure 1. The optimal pH for bioflocculant production was obtained at neutral pH (pH 7 ± 0.2). All subsequent experiments were conducted at pH 7 ± 0.2. A similar result was obtained for bioflocculant TKF04 production produced by *Citrobacter* spp. [30] and the increase in bioflocculant yield differs in different strains at their optimal pH [31]. The nutrient absorption capability of the cells and the presence of high electric charge could influence enzymatic reactions [32,33] thereby decreasing flocculating activity. At neutral pH, the nutrient absorption capability of the cells was high due to the neutral electric charge, indicating that the highest flocculation activity was observed in the broth culture.

#### 2.2.2. Effect of Inoculum Size

**Figure 2:**
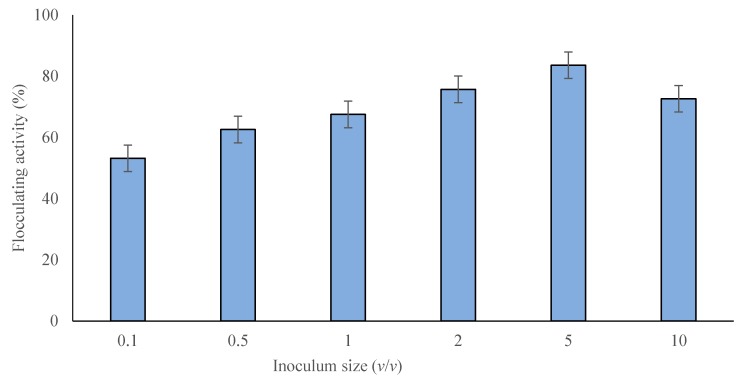
Effect of inoculum size on bioflocculant production.

The influence of inoculum volume on bioflocculant production was determined according to [34] using a range of bacterial inoculum sizes from 0.1% to 10% (*v*/*v*). The different inoculum volumes showed a certain effect on the flocculating activity and cell mass values, as shown in Figure 2. Flocculating activity increased from 53.2% at an inoculum size of 0.1% (*v*/*v*) to 83.6% at an inoculum size of 5% (*v*/*v*), followed by a decrease to 72.4% at an inoculum size of 10% (*v*/*v*). The obtained optimal inoculum size was 5% (*v*/*v*), which was used in all following experiments. The outcome results were analogous to those reported by Wang et al. [34] where an optimal inoculum volume of 5% was detected for *Klebsiella mobilis*. The maximum flocculation activity was not in agreement with the maximum cell growth, but the flocculation activity for *B. salmalaya* strain 139SI was maintained within a certain range of inoculum volume. This condition was due to the influence of inoculum volume on microbial growth. A small inoculum volume extended the lag phase growth, while outsized inoculum made the niche of the strain overlap extremely, subsequently inhibiting the production due to inadequate supply of nutrients [35].

#### 2.2.3. Effect of Temperature

**Figure 3:**
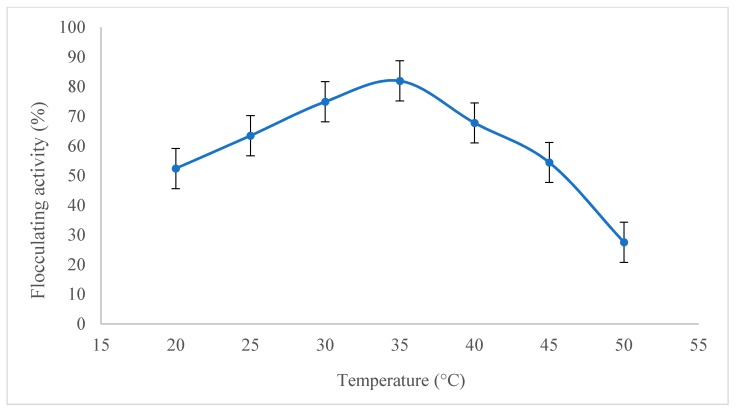
Effect of cultivation temperature on bioflocculant production.

The influence of temperature and shaking speed were used to investigate the bioflocculating activity of the bioflocculant. Figure 3 shows that the flocculating activity of bioflocculant was about 81.9%, when the culture temperature was at 35 °C, which was a best flocculating activity in the experiments. When the temperature was over 40 °C, the flocculating activity of the bioflocculant gradually declined. The optimal temperature for bioflocculant production was 37 °C, which was used for the following studies. The metabolism of microorganisms is directly related to culture temperature [2,32]. Maximum enzymatic activation can only be obtained at an optimum temperature [36].

#### 2.2.4. Effect of Shaking Speed

**Figure 4:**
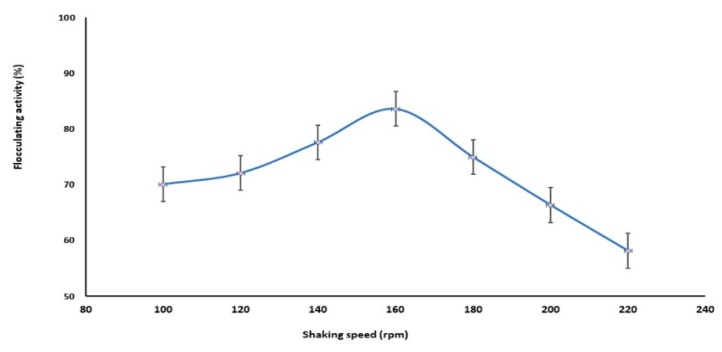
Effect of shaking speed on bioflocculant production.

The effect of shaking speed on bioflocculant production is shown in Figure 4. The optimum shaking speed was found to be 160 rpm, with highest flocculating activity reached 83.6%. Also, flocculating activity start gradually in an increase from 100 to 160 rpm. While higher shaking speed than the optimum caused a decline in the flocculating activity. The shaking speed of 160 rpm was used in the following experimental studies. The shaking speed determines the dissolved oxygen concentration, which can also affect nutrient absorption and enzymatic reaction [36]. In the course of the early growth phase, the biomass and bioflocculant production were lower, resulting in lower viscosity of culture broth and oxygen demand, when the strain *B. salmalaya* 139SI proceed in the logarithmic and stationary phases, the biomass and the bioflocculant production increased, and the corresponding viscosity of the culture broth and the oxygen demand also increased.

#### 2.2.5. Effect of Carbon Source

**Figure 5:**
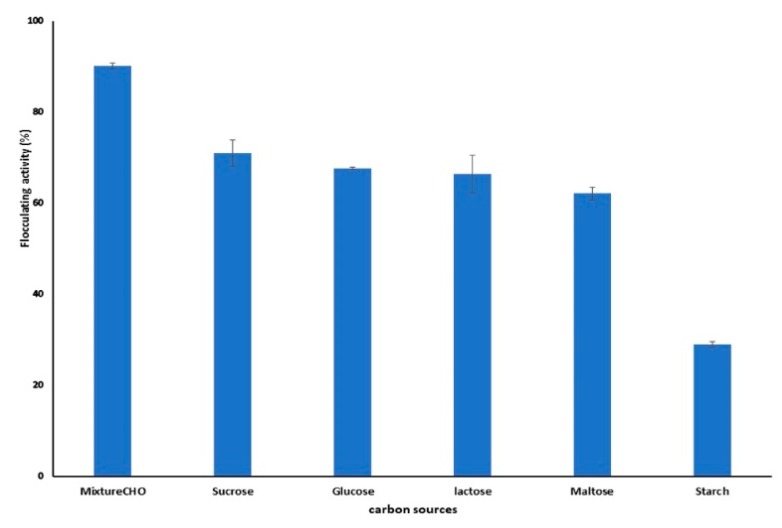
Effect of carbon source on bioflocculant production.

Bioflocculant synthesis is greatly affected by the type of carbon source used. The effect of carbon sources including sucrose glucose, lactose, maltose, fructose, and starch as single sources on bioflocculant synthesis by *B. salmalaya* strain 139SI was evaluated as shown in Figure 5. In addition, the bioflocculant synthesis was also tested under the effect of a mixture of carbohydrate (CHO) which includes sucrose and glucose. The highest 90.1% and the lowest 29% of flocculating activities were obtained with mixed carbohydrate source and starch, respectively. Moreover, as single carbon sources such as sucrose, glucose, lactose, and maltose were markedly appropriate for bioflocculant production with a flocculating activity exceeding 60% after 72 h of cultivation period. Aljuboori et al. [37] found that sucrose is the most desirable carbon source to *Aspergillus flavus* for bioflocculant production.

#### 2.2.6. Effect of Nitrogen Source

**Figure 6:**
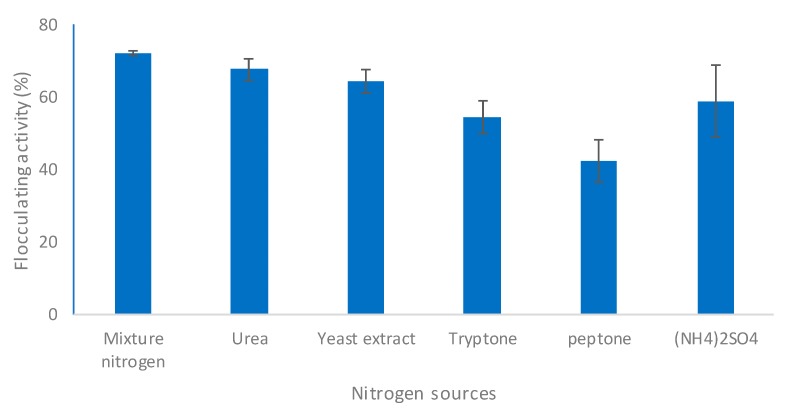
Effect of nitrogen source on bioflocculant production.

The nitrogen source plays a crucial role in the synthesis of bioflocculants [38]. The effect of nitrogen source on production was evaluated using different sources of organic origin, such as yeast extract, tryptone, peptone, urea, and ammonium sulfate was used as a source of inorganic nitrogen. In addition, the bioflocculant synthesis was also tested under the effect of a mixture of nitrogen which included urea and yeast extract. Different microorganisms utilize nitrogen derived from both or either inorganic or organic sources for production [39]. The influence of nitrogen sources, both organic and inorganic, on bioflocculant synthesis by *B. salmalaya* strain 139SI is illustrated in Figure 6. The highest flocculation activity reached at 72 h of cultivation was obtained with mixed nitrogen source 72.1%, while yeast extract gives 64.5% and urea 61.5%, whereas the lowest activity was detected with peptone 42.5%. While the ammonium sulfate as inorganic nitrogen gives about 58.86% of flocculating activity. As reported by Ugbenyen et al. [40], flocculating activity was peaked with yeast extract followed by casein hydrolysate and tryptone. A similar finding indicated that organic nitrogen is more appropriate for bioflocculant production than inorganic nitrogen because it is easily absorbed by microbial cells in relation to the nitrogen source of inorganic origin [38]. Moreover, Ismail and Nampoothiri [41] indicated that yeast extract was a highly favorable nitrogen source that improved EPS production by *Lactobacillus planetarium* MTCC 9510.

### 2.3. Bioflocculant Production by B. salmalaya Strain 139SI-7

**Figure 7:**
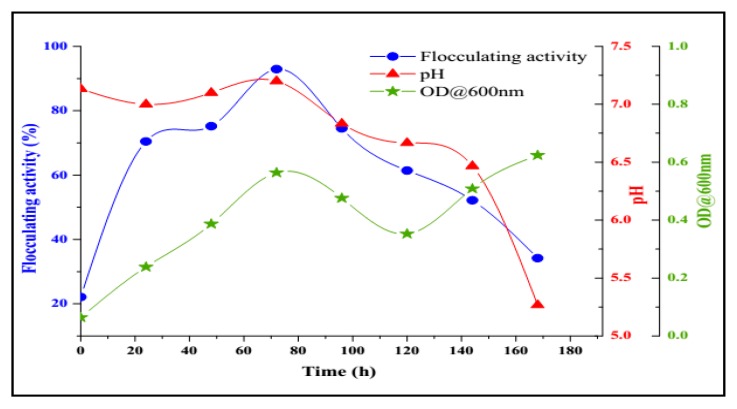
Time course of bioflocculant production by *Bacillus salmalaya* 139SI.

Optimal culture conditions were set for bioflocculant production by *B. salmalaya* 139SI. Figure 7 shows the time course assay of bioflocculant production. The produced amount of bioflocculant was 2.7 g/L.

As expected, no cell growth was observed within the first 12 h of cultivation (lag phase). However, after this period a steady increase in cell growth convoyed by a parallel increase in flocculating activity was detected. The stationary growth phase was attained after 72 h of cultivation. Flocculating activity ran parallel to cell growth, thereby exhibiting a concomitant increase in bioflocculant production with cell growth. Flocculating activity reached its maximum flocculation peaked at 92.6% at the late stationary phase (72 h), and a further expansion in cultivation period lead in a decrease in both flocculating activity and cell growth. This observation indicated that the production of bioflocculant was a result of biosynthesis during bacterial growth and not via cell autolysis [4]. The decrease in flocculating activity detected after 72 h might be attributed to the existence of bioflocculant-degrading enzymes produced by the microorganisms [42]. A similar study was reported by Zheng et al. 2008 [39], who found that the flocculating activity of the bioflocculant produced by *Serratia fiacre* and *Bacillus* sp. F19 reaches its maximum at the early stationary phase of 72 h. The initial pH of the production medium was adjusted to 7 and then monitored at regular intervals over the entire fermentation period. Moreover, the pH of the production medium governs the oxidation-reduction potential and the cells electrical charge thereby affecting enzymatic reaction and nutrient absorption [2]. Consequently, the pH of the fermentation medium decreased as cultivation period increased time. The decrease in pH of the fermentation medium might be due to the production of organic acids as a result of glucose metabolism, because glucose was a constituent of the production medium or the presence of organic acids metabolically produced by bacteria [43].

### 2.4. Characteristics of Bioflocculant QZ-7

The composition of bacterial bioflocculants plays a role in their flocculating activities [44] reports showed that numerous types of bioflocculants comprise proteins, polysaccharides, glycoproteins, and glycolipids [9]. On the basis of the chemical analysis of the produced bioflocculant, the total carbohydrate and protein compositions were 79.08% and 15.4%, respectively. These results are inconsistent with Chaplin and Kennedy [45]. The bioflocculant was mainly composed of polysaccharides and proteins. Further analysis revealed the presence of uranic acid in the bioflocculant. With an adequate proportion of uranic acid molecules in the bioflocculant, carboxyl groups can be added to the molecular chain. The carboxyl group present in the molecular sequence affords more active sites for constituent parts, so several elements can bind to the elongated molecular chain [46].

### 2.5. FTIR

**Figure 8:**
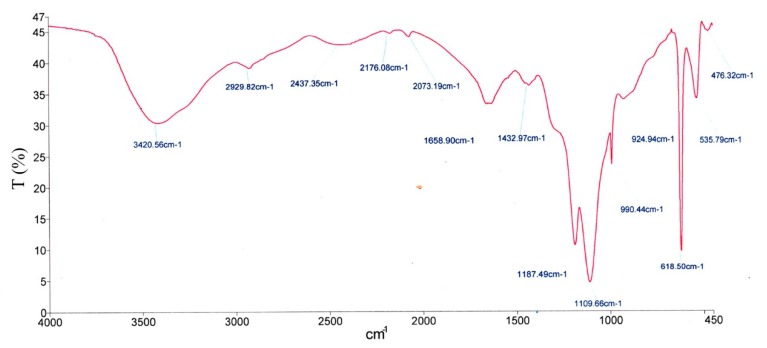
Fourier-transform infrared spectroscopy.

Infrared spectrophotometry was used to analyze the purity of the bioflocculant as illustrated in Figure 8. Clear absorption peaks were observed at 3420.56, 2929.82, 2437.35, 2176.08, 2073.19, 1658.90, 1432.97, 1187.49, 1109.66, 924.94, 618.50, 535.79, and 476.32 cm^−1^. The absorbed stretching O-H band was at 3420.56 cm^−1^, and a vibration weak band of C-H was noted at 2929.82 and 2073.19 cm^−1^. This result was similar to the results obtained by Deng 2005 [11]. The peaks at 1658.90 and 1432.97 cm^−1^ were attributed to C=O stretching and COOH vibration, respectively, whereas that at 1432.97 cm^−1^ was due to the C=O antisymmetric extension in the carboxylate [47], thereby showing the existence of carboxylate function groups in QZ-7. The carboxyl group may also serve as a functional moiety for the generation of modified or new polymers in different forms via different approaches, such as a unique designed formulation by assembling such polymers to other synthetic polymers. Other bands observed at 1109.66 and 1187.49 cm^−1^ were identified to be classic characteristics of all compounds derived from sugar, including sugar derivatives such as manuronic acid, guluronic acid, and uranic acid [48]. Other absorption bands at 924.94 and 990.44 cm^−1^ were related to the β-glycosidic bond linking the monomeric units present in sugars [49]. The corresponding small band absorptions were observed at 446.32, 538.79, and 618.50 cm^−1^, which indicated that the bioflocculant QZ-7 was a protein-bound polysaccharide [25].

### 2.6. Molecular Weight Analysis

The HPGPC spectrum of the purified bioflocculant exhibited a symmetrical and sharp peak in the retention time of 6.53 and 9.95. The molecular mass–retention time equation in accordance with the calibration curve was expressed as follows:

Log (molecular weight) = −0.1368T + 8.3496(1)

The average weight of the bioflocculant was calculated to be 5.13 × 10^5^ Da, which was much higher than the weight of other bioflocculants [50]. Bioflocculants with high molecular weight present stronger bridging, more adsorption points, and higher flocculating activities than those with low molecular weight [7].

### 2.7. SEM Imaging

**Figure 9:**
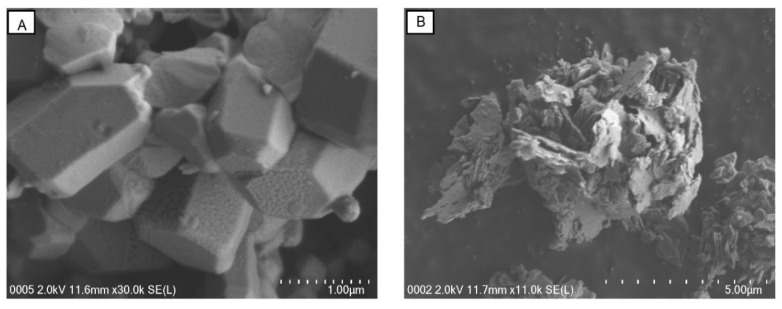
SEM micrograph, (**A**) purified bioflocculant QZ-7; (**B**) bioflocculant aggregation with kaolin clay.

The morphological surface structure of QZ-7 was illuminated prior to and after the flocculation process with kaolin clay particles. As shown in Figure 9A, QZ-7 was gray with a condensed crystalline brick-shaped structure. This structure served as an attachment site to which suspended particles and cations could bind [2]. Figure 9B illustrates how the bioflocculant aggregated the kaolin particles, which resulted in the formation of large flocs that were easily sedimented. Therefore, SEM images of QZ-7 and flocculating kaolin particles indicated that bridging could be liable for the flocculation capability of QZ-7. In accordance with our observation, previous studies also reported related incidences with some bioflocculants [24,51].

### 2.8. pH and Thermo-Stability of Pure Bioflocculant QZ-7

**Figure 10:**
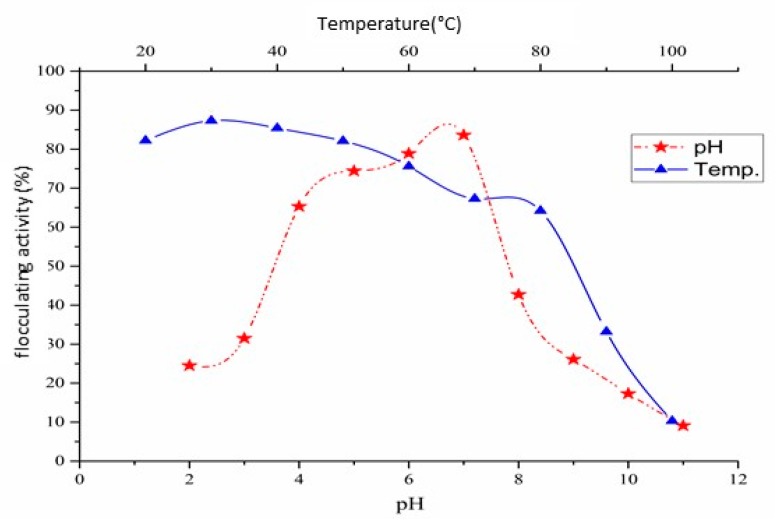
Thermal and pH stability of the purified QZ-7.

As shown in Figure 10, QZ-7 was found to be fairly steady at a wide pH range of 4–7, and about 85% flocculating activity was observed at this range. Therefore, QZ-7 was considered useful in neutral and acidic conditions, whereas a pH greater than 7 decreased the degree of flocculation activity. QZ-7 displays diverse electric statuses at varying pH ranges, and this characteristic may affect the flocculating capability of QZ-7 for kaolin units [52]. The influence of temperature on the flocculation activity of the purified QZ-7 was investigated. As shown in Figure 10, over 70% of flocculating activity was sustained at 20 °C–80 °C and pH 4–7. The thermal strength of this bioflocculant was attributed to the core backbone of QZ-7, which comprises polysaccharides [43]. The bioflocculant with polysaccharide-based structure are generally thermal-stable, but those with protein are sensitive to temperature [10,53]. High temperatures may result in the degradation of polysaccharide chains and diminish flocculating activity. The thermal activity of the biopolymer was investigated by other studies using different bioflocculants [2].

### 2.9. Wastewater Treatment with Bioflocculants

The crude bioflocculant produced by *B. salmalaya* 139SI was used to treat industrial wastewater with an initial COD concentration of 15,268 mg/L. After the treatment, the final COD concentration dropped to 1065 mg/L, and the COD removal rate was 93%. Therefore, the bioflocculant QZ-7 could be used for the removal of COD from wastewater. The *B. salmalaya* strain 139SI-7 was used to treat an industrial wastewater with an initial BOD concentration of 4018 mg/L. After treatment, the final BOD concentration was 302 mg/L. The percentage of BOD removal estimated by Equation (2) was 92.4%. Hence, *B. salmalaya* strain 139SI could be used for the removal of BOD from wastewater.

## 3. Conclusions

In this research, a production, optimization, and characterization of bioflocculant QZ-7 synthesized by a novel *Bacillus salmalaya* strain 139SI isolated from a private farm soil in Selangor, Malaysia, were conducted. Results show that the flocculating activity of bioflocculant QZ-7 present in the selected strain was found to be 83.3%. The optimal culture for flocculants production was achieved at pH 7 ± 0.2, with an inoculum size of 5% (*v*/*v*) and sucrose and yeast extract as carbon nitrogen sources after cultivation at 35 °C for 72 h. After optimization, the bioflocculant was increased by 10%. The maximum flocculating activity was found to be 92.6%. Chemical analysis revealed that the pure bioflocculant consisted of 79.08% carbohydrates and 15.4% proteins. The average molecular weight of the bioflocculant was calculated to be 5.13 × 10^5^ Da. Infrared spectrometric analysis showed the presence of carboxyl (COO-), hydroxyl (-OH), and amino (-NH3) groups; polysaccharides; and proteins. The bioflocculant QZ-7 exhibited wide pH stability ranging from 4 to 7, with a flocculation activity of 85% at pH 7 ± 0.2. In addition, QZ-7 was thermally stable and retained more than 80% of its flocculating activity after being heated at 80 °C for 30 min. After treating the wastewater, the bioflocculant QZ-7 showed significant flocculation performance with a COD removal efficiency of 93%, whereas a BOD removal efficiency of 92.4% was observed in the *B. salmalaya* strain 139SI. These values indicate the promising application of the bioflocculant QZ-7 in wastewater treatment.

## 4. Materials and Methods

### 4.1. Cultivation and Isolation of the Bacteria

The *B. salmalaya* strain 139SI was obtained from the Molecular Bacteriology and Toxicology laboratory at the University of Malaya. The bacterial isolate was originally obtained from soil samples obtained from a private farm located at 2.99917° N and 101.70778° E in Selangor, Malaysia. The bacterium was identified as *B. salmalaya* strain 139SI and deposited in Gen Bank KM0511837 [54]. The selected strain was streaked on blood agar medium plates and incubated at 37 °C for 18–24 h. The bacterial colonies exhibiting β-hemolytic activities were obtained and subcultured on slant tubes of nutrient agar. The subcultures were incubated under aerobic conditions at 37 °C for 24 h. This bacterial strain was consistently cultivated on nutrient agar and preserved in glycerol solution (20%, *w*/*v*) suspended at −80 °C.

### 4.2. Composition of the Used Media

Two types of media, namely, seed media and production media, were used to screen and obtain the produced bioflocculant. The seed medium had the following components (in g/L): 10 g of glucose, 1.5 g of yeast extract, 1.5 g of urea, 0.1 g of KH_2_PO_4_, 0.1 g of NaCl, and 0.2 g of MgSO_4_·7H_2_O. The pH was adjusted to 7.0 ± 0.2. The production medium contained the following components (in g/L): 10 g of sucrose, 5 g of glucose, 1.5 g of yeast extract, 1.5 g of urea, 0.1 g of KH_2_PO_4_, 0.1 g of K_2_HPO_4_, 0.1 g of NaCl, and 0.2 g of MgSO_4_·7H_2_O. The pH was adjusted to 7.0 ± 0.2 [55].

### 4.3. Screening for Bioflocculant-Producing Bacteria

Fifteen colonies of *B. salmalaya* strain 139SI were pre-cultured in 15 McCartney bottles each containing 10 mL of the production medium. The bottles were incubated at 35.5 °C and shaken at 150 rpm for 24 h. Subsequently, 2% of each culture broth was seeded into 100 mL of fermentation medium. The seeded flasks were also incubated at 35.5 °C for 24 h at 150 rpm. For cell separation, the fermented culture was harvested via centrifugation at 4000 rpm for 30 min [50].

### 4.4. Determination of Flocculating Activity

To select the best strain that produced bioflocculants, flocculating activity was determined from the cell-free supernatants. The flocculating activity was analyzed using a suspension of kaolin clay. The suspension was prepared by mixing 4.0 g of kaolin clay in 1.0 L of distilled water [56]. A mixture of 95 mL of kaolin suspension with 3 mL of 1.0% calcium chloride (CaCl_2_) solution and 2.0% (*v*/*v*) cell-free supernatant was prepared. The mixed solution was vigorously agitated and left to settle at room temperature for 5 min. The optical density (OD550) of the obtained clarified solutions was determined via spectrophotometry at 550 nm (UV-1700 spectrophotometer, Shimadzu, Kyoto, Japan). A control sample was prepared in the same way, except the cell-free supernatant was replaced with unfermented broth media. The flocculating activity was calculated using the following expression [56]:

Flocculating activity % = (A_c_ − B_s_)/A_c_ × 100 (2)

where A_c_ and B_s_ represent the OD of the control and real samples, respectively.

### 4.5. Optimization of Cultural Conditions for Bioflocculant Production

#### 4.5.1. Effect of pH

The effect of pH on the production medium was determined at different pH values ranging from 3–11 adjusted by adding 1 N HCl and 1 N NaOH as needed. A fresh culture of 2% (*v*/*v*) *B. salmalaya* strain 139SI-7 was inoculated into the prepared medium, incubated for 72 h at 35 °C, and shaken at 150 rpm. The flocculating activity was examined using kaolin clay to check the optimal pH required for bioflocculant production as indicated above [37].

#### 4.5.2. Effect of Inoculum Size

The influence of inoculum volume on bioflocculant production by *B. salmalaya* strain 139SI was examined because different inoculum sizes exert certain effects on the flocculation activity and cell mass growth. The inoculum sizes used were 0.1%, 0.5%, 1%, 2%, 5%, and 10% [37].

#### 4.5.3. Effect of Temperature and Shaking Speed

The *B. salmalaya* strain 139SI was inoculated into seed media and incubated at 37 °C on a shaker at 150 rpm for 24 h. From fresh culture of 2% *v*/*v*, was inoculated into several sets of 200 mL bottles containing 50 mL of production medium, then incubated at different cultivation temperatures were investigated, i.e., 25, 30, 35, 40 and 45 °C on shaking 150 rpm for 144 h. Also, the shaking speeds were investigated for different speeds such as 100, 120, 140, 160,180, 200 and 220 rpm, respectively. The cell free supernatant was obtained by centrifuge at 4000 rpm for 30 min to separate the cells. Then the flocculating activity was checked.

#### 4.5.4. Effect of Carbon and Nitrogen Sources

Bioflocculant production by microorganisms is significantly influenced by carbon and nitrogen sources [32]. These parameters were assessed according to Lachhwani [31]. The growth media were prepared in separate flasks. The bacterial strain was inoculated into the prepared medium. The media were supplemented with 10 g/L each of various carbon sources, incubated at 35.5 °C, and shaken at 150 rpm for 7 days. To determine the influence of nitrogen on bioflocculant production, 1.5 g/L each of various nitrogen sources was integrated into the fermentation medium in separate containers, and the flocculation activity was calculated according to Lachhwani using Equation (1) [31].

### 4.6. Bioflocculant Production by B. salmalaya Strain 139SI-7

Seed culture was prepared by inoculating 5% (*v*/*v*) of bacterial suspension in 50 mL of enriched medium, followed by overnight incubation at 35.5 °C and 160 rpm. For the optical density (OD600) test, sterile saline water was used to dilute the fermented broth to 0.1% [57]. In 500 mL of production medium, the optimized bacterial suspension was inoculated, incubated at 35.5 °C, and shaken at 160 rpm for 7 days. A 10 mL aliquot of the sample was collected periodically at timed intervals of 24 h, and 5 mL of the fermented broth was centrifuged. The obtained supernatant was used for the determination of bioflocculant activity following the methods of Kurane and Nohata [56]. The rate of bacterial growth was monitored via bacterial count using the standard plate method and OD_600_. The pH and flocculating activity were also determined during the study.

### 4.7. Extraction and Purification of Bioflocculants

At the end of the fermentation period, the culture was subjected to centrifugation for 15 min at 3500 rpm to separate pelleted bacterial cells. The extracted supernatant was mixed with one volume (*v*/*v*) of sterile distilled water, followed by centrifugation for 15 min at 3500 rpm to remove insoluble materials. Furthermore, the supernatant was mixed with two volumes of cold ethanol (1:2). The sample was thoroughly mixed with a stirrer and allowed to stand at 4 °C for 12 h. Subsequently, the precipitate was extracted. The obtained crude polymer was dissolved in sterile distilled water. The solution sample was then mixed with chloroform and n-butyl-alcohol in proportion (5:2, *v*/*v*) with stirring and allowed to stand at room temperature overnight. The upper surface portion was separated and subjected to centrifugation at 3500 rpm for 15 min to obtain a pure bioflocculant [3]. The purified supernatant was concentrated at 40 °C. To recover the precipitate, two volumes of ethanol were added. Finally, the precipitate was vacuumed, dried, dissolved in deionized water to obtain a pure bioflocculant (2.75 g/L) [38], and coded as bioflocculant QZ-7.

### 4.8. Characterization of the Bioflocculant QZ-7

#### 4.8.1. Chemical Analysis

Total carbohydrate concentration of the bioflocculant QZ-7 was assayed by the phenol-sulfuric acid method according to Chaplin and Kennedy [45], in which glucose solution was used to determine the standard curve. For uronic acid, the carbazole assay is the method used for detecting and quantifying free and polymeric uronic acids according to Chaplin and Kennedy [45]. The protein concentration was assayed using the Folin-Lowry method, in which bovine serum albumin served as a standard solution [31].

#### 4.8.2. pH Stability of Pure Bioflocculant QZ-7

The bioflocculant QZ-7 2 mg/mL was dissolved in 10 mL of deionized water to reach an initial flocculation activity above 85%. It was then separated into seven samples. The samples were adjusted to pH 4, 5, 6, 7, 8, 9, and 10 with 1 N HCl or 1 N NaOH. The samples were kept standing at 4 °C for 1 day [58]. Then, 4 g/L kaolin suspension was prepared and adjusted to pH 7 ± 0.2 the mixed solution was stirred 2 min at 220 rpm, then another round agitated for 10 min at 100 rpm and kept standing for 5 min. Flocculation activity was determined at room temperature.

#### 4.8.3. Thermo-Stability of Pure Bioflocculant QZ-7

Purified bioflocculant QZ-7 2 mg/mL was dissolved in 10 mL of distilled water to determine the optimum flocculation activity and then divided into five groups with pH 3, 5, 7, 9, and 11. The samples of each pH value were tested at 20 °C, 30 °C, 40 °C, 50 °C, 60 °C, 80 °C, and 100 °C for 60 min in a water bath [58]. The kaolin suspension (4 g/L) was adjusted to pH 7 ± 02, stirred for 2 min at 220 rpm, and agitated again at 100 rpm for 10 min, and allowed to stand for 5 min. The flocculation activity was determined at room temperature.

#### 4.8.4. Fourier-Transform Infrared Spectroscopy (FTIR)

The purified bioflocculant was further subjected to FTIR. The purified bioflocculant QZ-7 was blended with potassium bromide and compressed into a disc to obtain translucent pellets for FTIR analysis. The background reference compound used was the pelleted form of potassium bromide. Infrared absorption spectra were recorded with a Spectrum 400 instrument (PerkinElmer, Waltham Massachusetts (MA), USA). The spectral resolution and wave number accuracy were 400–4000 cm^−1^ under ambient conditions [59].

#### 4.8.5. Determination of Molecular Weight of Purified Bioflocculant

The molecular weight of QZ-7 was determined using a TSK G4000 PWXL column operated at 40 °C via high-performance gel permeation chromatography (HPGPC) coupled to a refractive index (RI) detector (Shimadzu). The column was calibrated using dextran standards. The mobile phase was deionized-distilled (DDI) water at a flow rate of 0.6 mL/min. Before injection, the sample was filtered through a 0.45 µm filter [55,60]. The following regression equation was obtained:

Log (mass) = K_1_T + K_2_ (3)

where mass (Da) and T (min) are the molecular mass and retention time of the samples, respectively, and K_1_ and K_2_ are constants.

#### 4.8.6. Scanning Electron Microscopy (SEM) Remarks

The SEM remarks were examined. The purified bioflocculant QZ-7 powder was spread and fixed on the iron stub. The fixed sample was scanned using SEM (HITACHI- SU8220, Tokyo, Japan).

### 4.9. Removal of COD from Wastewater with Bioflocculant QZ-7

Approximately 0.3 g of the prepared bioflocculant QZ-7 was added to 100 mL of filtered wastewater. The physio-chemical characteristics of the raw wastewater were; 4018 mg/L BOD, 15,268 mg/L COD, 17,832 mg/L TDS, and 500 NTU color. The system was agitated with a magnetic stirrer at room temperature for 2 min at 200 rpm and for 10 min at 50 rpm. The samples were left to stand for 15 min and clarified through a 0.45 µm membrane filter paper. The final COD was determined via the closed reflux colorimetric method [61] The COD removal rate was calculated using the following equation:

R = [(COD_i_ − COD_f_)/COD_i_] × 100 (4)

where R is the removal rate in %, COD_i_ is the COD concentration of the wastewater before treatment, and COD_f_ is the COD concentration of the wastewater after treatment.

### 4.10. Removal of BOD from Wastewater with B. salmalaya Strain 139SI-7

The removal of BOD from wastewater with *B. salmalaya* strain 139SI was carried out using two diluted samples. The initial dissolved oxygen (DO_i_) of the first sample was measured using a DO meter. The other sample was inoculated with 10% of the bacterial suspension and incubated in a BOD incubator for 5 days. The final dissolved oxygen (DO_f_) was measured after 5 days. The BOD was calculated using Equation (4) [61] and the BOD removal percentage was calculated using the following equation:

BOD (mg/L) = DO_i_ − DO_f_ (5)

## Figures and Tables

**Table 1:** Flocculating activity values for the selected strains.

Strain Code No	Flocculating Activity (%)	Standard Deviation (SD)
BS * 139SI-1	67.5	0.655
BS 139SI-5	54.2	0.770
BS 139SI-7	83.3	0.75
BS 139SI-8	72.2	1.93
BS 139SI-13	63.4	0.45

* BS is *Bacillus salmalaya*.
